# Corticosterone as a Physiological Biomarker: Decoding the Environment‐Cort‐Energy Paradigm

**DOI:** 10.1002/ece3.73572

**Published:** 2026-04-29

**Authors:** B. Sunny Domschot, Thomas V. Riecke, Jessica L. Malisch, Thomas P. Hahn, Creagh W. Breuner

**Affiliations:** ^1^ Wildlife Biology Program The University of Montana Missoula Montana USA; ^2^ University of California Merced Natural Reserve System Merced California USA; ^3^ Neurobiology, Physiology, and Behavior University of California Davis Davis California USA; ^4^ Ecology and Evolution Program University of Montana Missoula Montana USA

**Keywords:** climate, corticosteroid‐binding‐globulin, corticosterone regulation, glucocorticoid variation, sex‐specific stress responses, structural equation modeling in ecology

## Abstract

Glucocorticoid hormones (GCs) are widely used to assess physiological responses to environmental variation in wild animals, yet uncertainty remains over which circulating measures best reflect biologically meaningful stress responses. Total plasma GCs are most commonly measured across vertebrates. However, the fraction reaching tissues—the free hormone—is regulated by the plasma binding protein corticosteroid binding globulin (CBG). We examined corticosterone dynamics in a free‐living songbird, the mountain white‐crowned sparrow ( *Zonotrichia leucophrys oriantha* ), using 14 years of repeated physiological, morphological, and environmental measurements. We applied structural equation modeling to compare three conceptual frameworks describing plasma glucocorticoid physiology: models based on total circulating GCs, free GCs (unbound to CBG), and both total GCs with CBG variation. Across models, we identified links between environmental conditions, energetic state, and GC/CBG physiology, but the strength and structure of these relationships differed by sex and by the hormonal measure considered. Models examining relationships with free hormone included a greater number of supported explanatory variables than models based on total hormone alone, while models incorporating CBG dynamics provided additional explanatory structure and clarified sex‐specific patterns not fully captured by either measure individually. Our results indicate that inferences about glucocorticoid–environment relationships depend strongly on how hormone physiology is characterized, highlighting the importance of integrating binding dynamics and sex‐specific physiology when interpreting glucocorticoid variation.

## Graphical abstract

Corticosterone (cort) is increasingly used as a biomarker in wildlife conservation, though debate remains over which measures best capture physiological and environmental relationships. Using structural equation modeling on a 14‐year dataset, we evaluated the total hormone, free hormone, and CBG profile hypotheses linking cort, energetic state, and environmental factors. The CBG profile model provided the most comprehensive, sex‐specific insights, emphasizing the importance of selecting appropriate cort measures to enhance physiological interpretation and conservation applications.

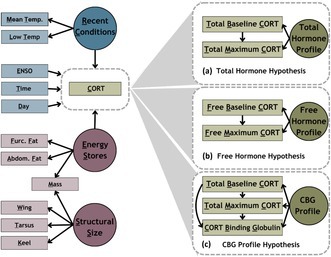

## 1 Introduction

Glucocorticoid (GC) hormones play a central role in mediating physiological and behavioral responses to environmental variation across vertebrates (Romero and Wingfield 2016). GCs cycle at low, baseline levels on a diel basis, binding to high affinity mineralocorticoid receptors in the brain to regulate metabolism. These levels increase in response to stressors acting through low affinity glucocorticoid receptors (Reul et al. 1987). This spike in secretion facilitates a range of behavioral and physiological changes that enable organisms to better cope with challenges (Wingfield et al. 1998; Crespi et al. 2013; Lattin et al. 2016). However, chronically elevated levels can reduce an animal's fitness (Sapolsky et al. 2000). Measures of these hormones can therefore provide information on an individual's coping strategy in response to environmental change and human disturbance (Wingfield et al. 1998). Despite their widespread use in ecological research (Madliger et al. 2018), interpreting variation in GC levels remains challenging due to the complexity of endocrine regulation and the diversity of mechanisms through which GCs act (Sapolsky et al. 2000; Dantzer et al. 2014).

Measuring GCs in free‐living animals offers several benefits. First, GC measurements can relate environmental drivers (such as climate change or human disturbance) to fitness metrics, and the plasticity of this response can describe an individual's tolerance toward change (Seebacher and Franklin 2012). Second, these measures can reflect both acute and chronic responses to stress, providing insight into whether individuals are responding in healthy and sustainable ways (Busch and Hayward 2009). Finally, GC levels can be measured in non‐invasive ways (e.g., from feces, feathers, or hair), reducing additional disturbance costs of capture and handling (Cooke et al. 2013; Cooke and O'Connor 2010). However, before we can use GC measures to their full potential in monitoring wildlife health, clarification is needed regarding species‐ and context‐specific predictors of GC variation and the GC measures most appropriate for different research aims.

Across vertebrates, GCs are secreted via the hypothalamic–pituitary–adrenal (HPA) axis in birds, mammals, and reptiles (Denver 2009), and via the hypothalamic–pituitary‐interrenal (HPI) axis in fish and amphibians (Wendelaar Bonga 1997). While cortisol is the primary circulating GC in many mammals and fish, corticosterone (cort) predominates in birds, reptiles, and amphibians. In plasma, these hormones exist either bound to corticosteroid‐binding globulin (CBG), a plasma protein responsible for transport and delivery, or unbound by CBG and therefore free to enter tissues. GCs reversibly bind to CBG according to physiological and environmental conditions (Hadley and Levine 2007). When GCs are bound by CBG in the plasma, their availability and delivery to tissues is highly regulated by the capacity, affinity, and specificity of an organism's CBG (Breuner et al. 2006; Breuner et al. 2020). Since CBG binds a significant portion of circulating GCs (Rosner 1990), changes in CBG can alter the amount of unbound hormone and therefore its biological effects (Mendel 1992; Perogamvros et al. 2011), adding an additional layer of complexity to interpreting GC variation.

Individual GC levels further vary with environmental and physiological conditions, increasing variability across individuals and habitats and complicating inference between GCs and health metrics (Breuner et al. 2008; Bonier et al. 2009). One important source of such environmental variation is large‐scale climate oscillations such as the El Niño–Southern Oscillation (ENSO), which generate interannual variation in temperature, precipitation, and snowpack, influencing breeding phenology and resource availability for wildlife populations (Trenberth 1997; Stenseth et al. 2002). Sex‐specific differences in reproductive timing, energetic demands, and endocrine regulation further complicate interpretation of GC measures (Hau et al. 2010), as males and females may rely on distinct physiological mechanisms to achieve similar functional outcomes. As a result, interpreting hormone‐environment relationships requires careful consideration of both environmental drivers and sex‐specific physiological regulation (Breuner, Delehanty, and Boonstra 2013).

These sources of physiological and environmental complexity have led to several competing hypotheses regarding which measure of GCs–total, free, or bound to CBG– is a better explanatory variable when studying the hormone's action, with each having important implications for the mechanisms that may drive an organism's endocrine plasticity in response to stressors. The “Total Hormone” hypothesis suggests that total GC concentrations are the biologically relevant measure of the stress response (Breuner, Delehanty, and Boonstra 2013; Schoech et al. 2013). Under this hypothesis, it is assumed that both free and CBG‐bound hormone in the plasma can enter target tissues, likely because GCs can dissociate from CBG rapidly enough (with a high off‐rate) that binding does not limit tissue entry (Breuner, Delehanty, and Boonstra 2013; Breuner and Orchinik 2002). In contrast, the “Free Hormone” hypothesis (Mendel 1989) posits that only unbound, free GCs are biologically active. Under this hypothesis, CBG‐bound GCs in the plasma are considered functionally inactive, and only the free fraction is available to enter target tissues and elicit physiological responses (Mendel 1989; Hammond 2016). Finally, here we introduce the “CBG Profile” hypothesis, inspired by the “Reservoir Hormone” hypothesis (Malisch and Breuner 2010), which proposes that CBG‐bound hormone serves as a dynamic reservoir that modulates hormone availability to tissues. Under this hypothesis, only free GCs are available to target tissues, but CBG plays a crucial regulatory role by controlling the proportion of hormone that is biologically available (Breuner and Orchinik 2002; Malisch and Breuner 2010). Therefore, the dynamic regulation of CBG relative to total hormone concentrations could be a key mechanism by which individuals maintain homeostasis and effective stress responses (Breuner, Delehanty, and Boonstra 2013). Support for these hypotheses varies across taxa and contexts, and each approach provides valuable but incomplete information (Breuner et al. 2008; Bonier et al. 2009). Clarifying the relationships among factors that influence biologically active GC levels is therefore essential for building informative models and improving interpretation of cort physiology in conservation and ecological contexts (Madliger et al. 2018). Together, these competing hypotheses highlight the need for integrative approaches that can simultaneously evaluate multiple components of GC physiology and their relationships with environmental and physiological drivers (Arhonditsis et al. 2006; Grace et al. 2010).

Here we develop and present a flexible structural equation modeling (SEM) approach for a more robust analysis of a powerful physiological biomarker—corticosterone (cort) and apply it to a long‐term data set to assess the relationships between environmental and energetic variables and different cort measures. We use a 14‐year data set collected on Mountain White‐crowned Sparrows ( *Zonotrichia leucophrys oriantha* ) breeding in the central high Sierra Nevada, just outside of Yosemite National Park, CA, where interannual variation in snowpack and breeding conditions is strongly influenced by ENSO dynamics. Through this analysis, we aim to provide a generalizable framework for conservation scientists to use in determining which cort and covariate measures are relevant to collect in their study system, and to demonstrate how integrative modeling approaches can improve interpretation of physiological data.

## 2 Materials and Methods

### 2.1 Field Methods and Data Acquisition

Mountain white‐crowned sparrows breed in sub‐alpine meadows across the Western United States. The focal population for this study breeds at an elevation of 3000 m, just outside Yosemite National Park in the Sierra Nevada (37.8° N, 119.2° W) and has been studied nearly continuously since 1968. The high Sierra Nevada experiences widely variable winter snowpack accumulation depending on the Southern Oscillation Current (Redmond et al. 1991; Cayan 1996). White‐crowned sparrows in this population lay eggs as snow cover reaches approximately 50%, leading to substantial inter‐annual variation in reproductive timing (Morton 1976, 2002).

Sampling occurred almost yearly during May from 2001 to 2019. Individuals were captured and sampled multiple times both within and across years, resulting in repeated measures for many individuals. At each capture, individuals were sampled over 30 min to quantify the cort response to standardized capture and handling stress. Blood samples were collected within 3 min of capture (baseline) and again at 15 and 30 min after capture. For each capture event, maximum cort was defined as the highest cort concentration observed among the three samples, typically occurring at either 15 or 30 min post‐capture. See Patterson et al. (2014) for a more detailed description. Morphological measures (wing chord, tarsus, keel, mass) and fat score were taken after the 30‐min blood sample. This dataset is unique in both its duration (14 years: 2001–2010, 2016–2019) and its number of cort observations (456 individuals; 910 capture events; 1739 plasma cort samples). Because individuals were sampled unevenly across years, the number of capture events per individual varied, with some individuals contributing multiple repeated measurements and others sampled fewer times. This imbalance was explicitly accounted for in our structural equation models by treating individual identity as a grouping structure and modeling each capture event as a nested observation, allowing individuals with differing numbers of recaptures to contribute proportionally to parameter estimation without requiring separate models for different recapture frequencies. All hormone and CBG samples were assayed as per Patterson et al. (2014). Assay parameters (detectability and intra‐assay CoV) from this population covering much of the data incorporated here have been reported across 6 different studies (Breuner and Hahn 2003; Breuner et al. 2006; Lynn et al. 2003; Crino et al. 2011; Crino et al. 2013; Breuner, Delehanty, and Boonstra 2013), with an average detectability of 1.98 ng/mL (range 0.5–5), and an average intra‐assay variation of 8.4% (range 5%–15%).

Barometric pressure was measured in Tuolumne Meadows, Yosemite National Park (37.9° N, 119.4° W, within 15 km of the study site) by Dr. Jessica Lundquist as part of a long‐term environmental study. The barometric pressure was logged every 30 min from 2001 to 2015. Temperature data were collected using in‐field loggers (2001–2006) and supplemented with gridded climate data from the PRISM Climate Group (2014) at Oregon State University (2007–2019).

We used the Oceanic Niño Index (ONI) for El Niño–Southern Oscillation (ENSO) metrics. The ONI tracks the running 3‐month average sea surface temperature compared to a 30‐year average to provide anomaly index values. We interpreted index values of +0.5 or higher as indicating El Niño episodes, which result in wetter‐than‐usual conditions in the high Sierra (more snowfall = later nest initiation; Morton 1976, 2002). Conversely, we interpreted index values of −0.5 or lower as indicating La Niña episodes, which correlate with drier‐than‐usual conditions (NOAA 2026). Percent snow cover was estimated at the field site sporadically throughout May, but data were too sparse to use directly in the models. ONI values from March–April–May (MAM), however, were positively associated with percent snow cover at the field site (*β* = 0.056 ± 0.016 SE, *p* < 0.001), with the magnitude of this relationship (*R* ^2^ = 0.019) consistent with expectations for large‐scale climate indices influencing local environmental conditions (Stenseth et al. 2002). Therefore, we used MAM values for analyses.

### 2.2 Model Specification

**FIGURE 1:**
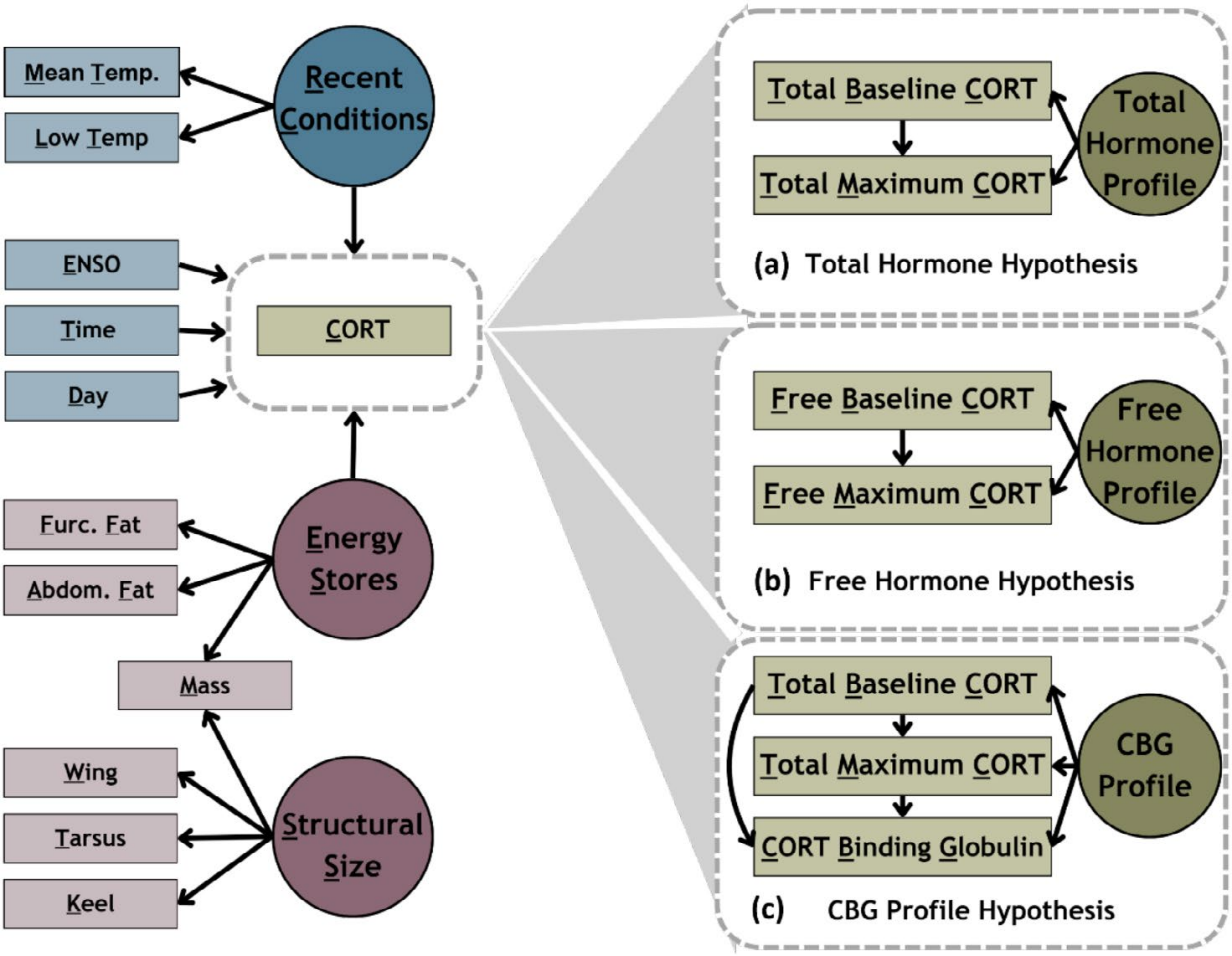
Causal Diagram of hypothesized relationships between energy stores, environmental conditions and cort levels for the (a) total hormone, (b) free hormone, and (c) CBG profile hypotheses. Measured variables are represented by solid boxes, latent variables are represented by circles, and the cort submodels for each hypothesis are represented by dashed boxes.

We developed three structural equation models (SEMs) in a hierarchical Bayesian framework (Grace et al. 2010) to analyze the relationships among environmental conditions, energetic stores, and cort measurements and assess three distinct hypotheses: the total hormone hypothesis (long‐standing but first named in Breuner and Orchinik 2002), the free hormone hypothesis (Mendel 1989), and the CBG profile hypothesis (Figure 1).

#### 2.2.1 Recent Conditions

We assessed the influence of local environmental factors—changes in mean temperature (*mt*) and barometric pressure (*bp*) over the previous 24 h (Breuner et al. 2013)—on cort levels using a latent variable representing these environmental parameters. We used factor loadings within the SEM framework to estimate the effect of these factors on cort levels.

$${\mathrm{RC}}_{i}\sim \text{Normal}\left(0,\sigma_{\mathrm{RC}}^{2}\right)$$

$$\sigma_{\mathrm{RC}}\sim \text{Gamma}\left(3,1\right)$$

$${\mathrm{mt}}_{i}\sim \text{Normal}\left(\alpha_{\mathrm{MT}}+\lambda_{{\mathrm{RC}}_{1}}{\mathrm{RC}}_{i},\sigma_{\mathrm{MT}}^{2}\right)$$

$${\mathrm{bp}}_{i}\sim \text{Normal}\left(\alpha_{\mathrm{BP}}+\lambda_{{\mathrm{RC}}_{2}}{\mathrm{RC}}_{i},\sigma_{\mathrm{BP}}^{2}\right)$$

#### 2.2.2 Energetic Stores

We modeled energetic stores as a latent variable comprised of furcular fat, abdominal fat, and size‐corrected mass with variance:

$${\mathrm{ES}}_{i}\sim \text{Normal}\left(0,\sigma_{{\mathrm{ES}}_{j}}^{2}\right)$$

$$\sigma_{{\mathrm{ES}}_{j}}\sim \text{Gamma}\left(1,1\right)$$

We defined furcular fat (*ff*) and abdominal fat (*af*) as normally distributed around a sex‐specific mean with variance,

$${\mathrm{ff}}_{i}\sim \text{Normal}\left(\alpha_{{\mathrm{FF}}_{j}}+\lambda_{{\mathrm{ES}}_{1,j}}{\mathrm{ES}}_{i},\sigma_{\mathrm{FF}}^{2}\right)$$

$${\mathrm{af}}_{i}\sim \text{Normal}\left(\alpha_{{\mathrm{AF}}_{j}}+\lambda_{{\mathrm{ES}}_{2,j}}{\mathrm{ES}}_{i},\sigma_{\mathrm{AF}}^{2}\right)$$

Mass (*m*) was modeled as normally distributed around a sex‐specific mean with an additional effect of structural size,

$$m_{i}\sim \text{Normal}\left(\alpha_{M_{j}}+\lambda_{{\mathrm{SS}}_{4,j}}{\mathrm{SS}}_{i}+\lambda_{{\mathrm{ES}}_{3,j}}{\mathrm{ES}}_{i},\sigma_{M}^{2}\right)$$

We modeled structural size as a latent variable measured via keel (*k*), wing chord (*w*), and tarsus length (*tl*) and treated repeated measurements within individuals as measurements with errors around an individual‐specific mean. To account for sex differences, we estimated separate parameters for females and males.

$${\mathrm{SS}}_{i}\sim \text{Normal}\left(0,\sigma_{{\mathrm{SS}}_{j}}^{2}\right)$$

$$\sigma_{{\mathrm{SS}}_{j}}\sim \text{Gamma}\left(1,1\right)$$

$$k_{i}\sim \text{Normal}\left(\alpha_{K_{j}}+\lambda_{{\mathrm{SS}}_{1,j}}{\mathrm{SS}}_{i},\sigma_{K}^{2}\right)$$

$$w_{i}\sim \text{Normal}\left(\alpha_{W_{j}}+\lambda_{{\mathrm{SS}}_{2,j}}{\mathrm{SS}}_{i},\sigma_{W}^{2}\right)$$

$${\mathrm{tl}}_{i}\sim \text{Normal}\left(\alpha_{{\mathrm{TL}}_{j}}+\lambda_{{\mathrm{SS}}_{3,j}}{\mathrm{SS}}_{i},\sigma_{\mathrm{TL}}^{2}\right)$$

#### 2.2.3 Temporal Variation

We used standardized time since sunrise of sample (*t*) to control for fluctuations in cort levels that happen naturally over a day regardless of stressors and other factors (Breuner et al. 1999). We used standardized day in May of sample (*d*) to control for seasonal variations in cort dynamics (Breuner and Orchinik 2001; Romero 2002; Cornelius et al. 2011).

#### 2.2.4 Cort

For each hypothesis, we modeled true underlying cort measurements ($C_{i}$) around an individual‐specific mean with sex‐specific effects of (1) energetic stores (${\mathrm{ES}}_{i}$), (3) recent conditions (${\mathrm{RC}}_{i}$), (4) El Niño–Southern Oscillation (${\text{ENSO}}_{i}$), and (5) time ($T_{i}$) and (6) day ($D_{i}$) of sample.

To assess the “Total Hormone Profile” (Figure 1a), we modeled repeated baseline ($C_{B,i}$) and maximum cort measures ($C_{M,i}$) within individuals as measurements around an individual‐specific mean with variance,

$$C_{B,i}\sim \text{Normal}\left(\mu_{B,\mathrm{ind}}+\beta_{1,j}{\mathrm{ES}}_{i}+\beta_{2,j}{\mathrm{RC}}_{i}+\beta_{3,j}{\text{ENSO}}_{i}+\beta_{4,j}T_{i}+\beta_{5,j}D_{i},\sigma_{C,B}^{2}\right)$$

with an additional effect of baseline measures on response measures,

$$\begin{array}{c} C_{M,i}\sim \\ \text{Normal}\left(\mu_{M,\mathrm{ind}}+\beta_{6,j}{\mathrm{ES}}_{i}+\beta_{7,j}{\mathrm{RC}}_{i}+\beta_{8,j}{\text{ENSO}}_{i}+\beta_{9,j}T_{i}+\beta_{10,j}D_{i}+\beta_{11,j}C_{B,i},\sigma_{C,M}^{2}\right) \end{array}$$

We tested the “Free Hormone Profile” (Figure 1b) using the same equations, where the response variables were free measures of cort rather than total cort. Finally, for the “CBG Profile” model (Figure 1c), we additionally modeled CBG measures ($C_{\mathrm{CBG},i}$) within individuals as measurements around an individual‐specific mean with variance, with additional effects of both baseline and maximum measures,

$$\begin{array}{c} C_{\mathrm{CBG},i}\sim \text{Normal}\left(\mu_{\mathrm{CBG},\mathrm{ind}}+\beta_{12,j}{\mathrm{ES}}_{i}+\beta_{13,j}{\mathrm{RC}}_{i}+\beta_{14,j}{\text{ENSO}}_{i}+\beta_{15,j}T_{i}\right.\\ \left.+\beta_{16,j}D_{i}+\beta_{17,j}C_{B,i}+\beta_{18,j}C_{M,i}\sigma_{\mathrm{CBG}}^{2}\right) \end{array}$$

where

$$\beta_{j}\sim \text{Normal}\left(0,1\right)$$

$$\sigma_{C}\sim \text{Gamma}\left(1,1\right)$$

#### 2.2.5 Computational Details

All analyses were conducted in R (R Core Team, 2020) and JAGS (Plummer 2003) using the jagsUI package (Kellner 2016). We sampled three MCMC chains for 150,000 iterations and discarded the first 20,000 iterations. We ensured all posterior distributions had values < 1.01 (Brooks and Gelman 1998) and visually inspected trace plots for convergence (Kéry and Schaub 2012). In the following text, tables, and figures, we report medians of posterior distributions, 95% Bayesian credible intervals, and *υ*, the proportion of the posterior distribution on the same side of zero as the mean. We assessed the strength of evidence for effects as follows: relationships where > 85% of the posterior distribution was on the same side of zero as the mean were regarded as providing some evidence, and relationships where > 95% of the posterior distribution was on the same side of zero as the mean were regarded as providing strong evidence.

## 3 Results

### 3.1 The Total Hormone Profile Model

**FIGURE 2:**
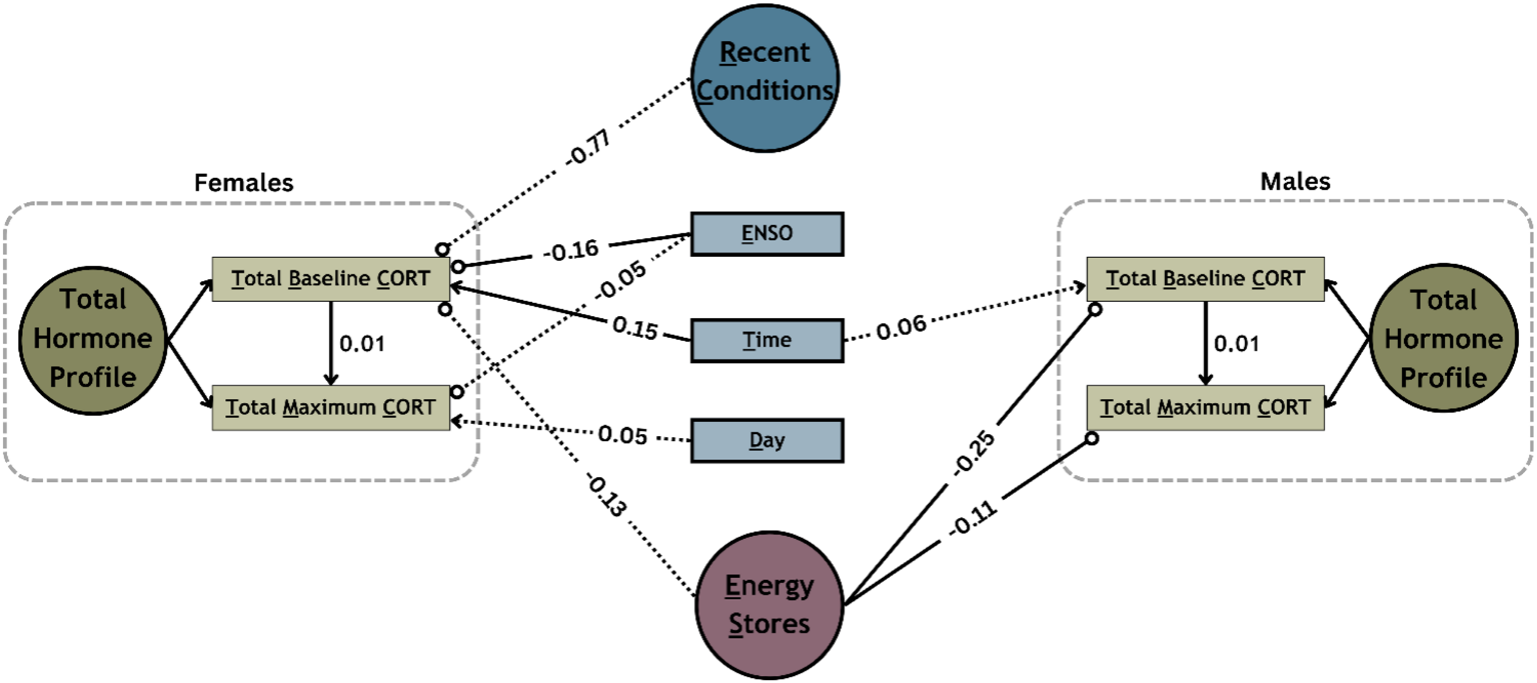
The total hormone profile: mean effects of environmental (recent conditions & ENSO) and energetic (Energy Stores) variables for both females and males. Significant positive (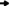) and negative (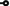) effects with ≥ 95% (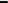) and ≥ 85% (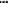) confidence.

The “Total Hormone Profile” model (Figure 1a) evaluated the relationships between environmental and energetic variables and total cort measures as the biologically relevant response measure. From this model, we found only a few strong relationships for either sex (Figure 2; Table A1). For Total Baseline Cort (TBC), we found strong evidence of a negative effect of ES (Energetic Stores, a latent variable derived from abdominal fat, furcular fat, and size‐corrected mass) in males (*β* = −0.25; *υ* = 1), and some evidence of a negative effect in females (*β* = −0.13; *υ* = 0.92), suggesting that individuals in better energetic condition tend to have lower TBC. Additionally, in females, we found strong evidence of a negative effect of ENSO (*β* = −0.16; *υ* = 0.99) on TBC, indicating lower levels during wet ENSO years. For Total Maximum Cort (TMC) in males, we found strong evidence for a negative effect of ES (*β* = −0.11; *υ* = 1), again indicating that better energetic condition is associated with lower levels. For female TMC, there was some evidence for a negative effect of ENSO (*β* = −0.05; *υ* = 0.90), again pointing to lower levels during wet ENSO years.

### 3.2 The Free Hormone Profile Model

**FIGURE 3:**
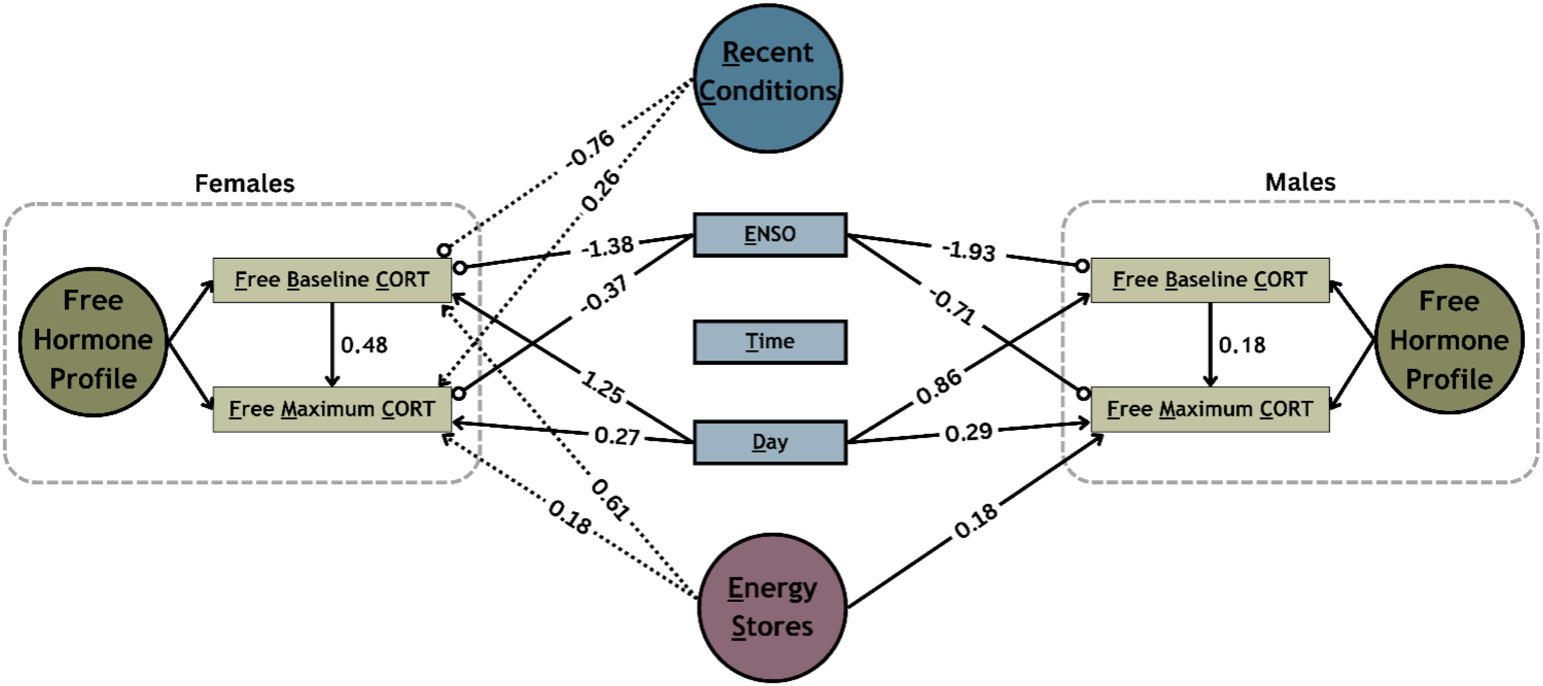
Free hormone profile: mean effects of environmental (recent conditions & ENSO) and energetic (Energy Stores) variables for both females and males. Significant positive (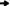) and negative (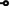) effects with ≥ 95% (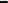) and ≥ 85% (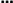) confidence.

The “Free Hormone Profile” model (Figure 1b) evaluated relationships between environmental and energetic variables and free baseline and maximum cort levels using estimates of unbound cort as the biologically relevant response measure. From the free hormone profile model, we found several strong relationships between both environmental and energetic variables and free cort measures (Figure 3; Table A2). For Free Baseline Cort (FBC), we found strong evidence for a negative effect of ENSO in both males (*β* = −1.93; *υ* = 1) and females (*β* = −1.38; *υ* = 1), consistent with lower FBC during El Niño years when breeding conditions are less favorable (wet). Additionally, we found some evidence for a positive effect of energetic stores (ES) in females (*β* = 0.61; *υ* = 0.85), indicating higher FBC with improved energetic condition, and a negative effect of recent conditions (RC) (*β* = −0.76; *υ* = 0.85), indicating lower FBC with improved recent conditions. For Free Maximum Cort (FMC), we found strong evidence for a positive effect of ES for males (*β* = 0.18; *υ* = 0.97), suggesting higher FMC for individuals in better energetic condition. We also found some evidence for a positive effect of RC on FMC in females (*β* = 0.26; *υ* = 0.85), indicating higher levels with improved recent conditions and strong evidence for a negative effect of ENSO on FMC in both males (*β* = −0.71; *υ* = 1) and females (*β* = −0.37; *υ* = 0.99), suggesting lower FMC during unfavorable El Niño years.

### 3.3 The CBG Profile Model

**FIGURE 4:**
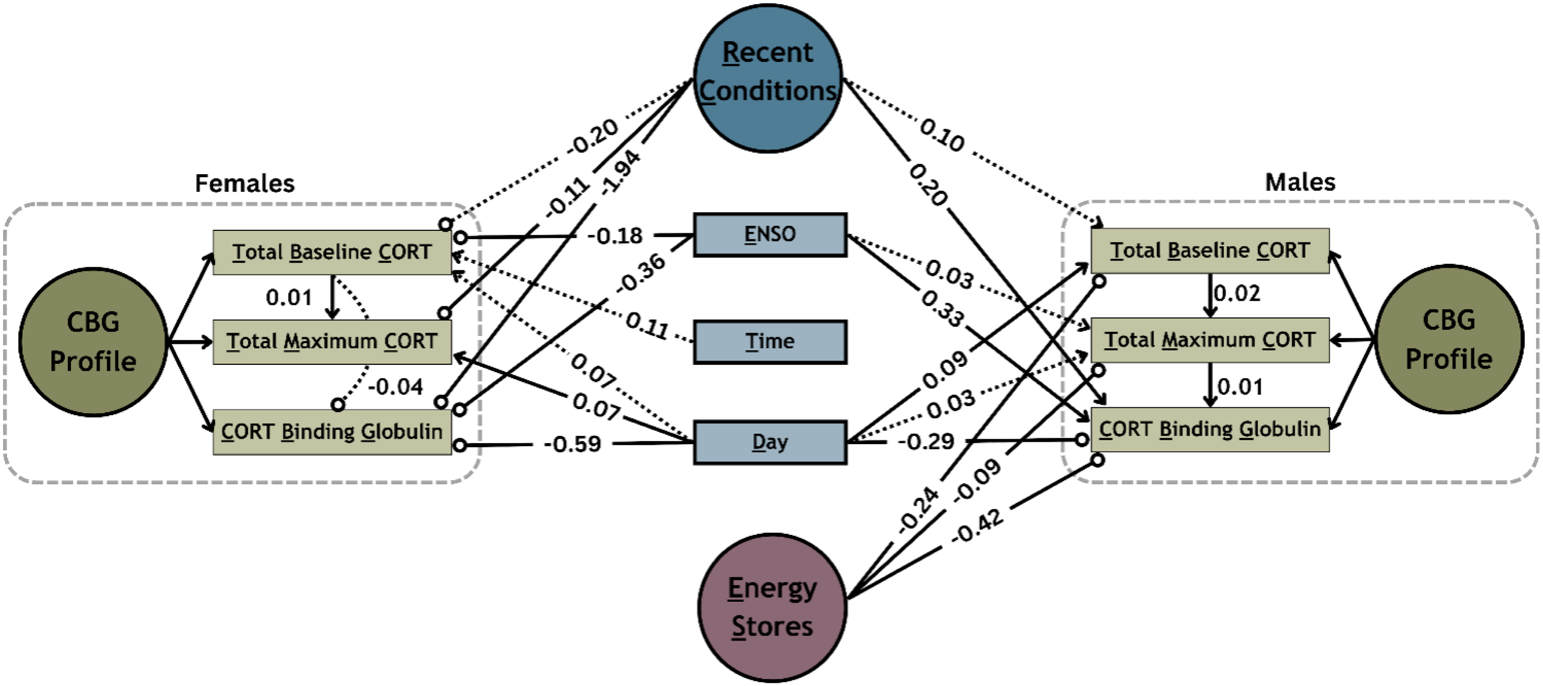
CBG profile: mean effects of environmental (recent conditions & ENSO) and energetic (Energy Stores) variables for both females and males. Significant positive (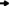) and negative (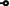) effects with ≥ 95% (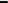) and ≥ 85% (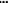) confidence.

The “CBG Profile” model (Figure 1c) evaluated relationships between environmental and energetic variables and CBG levels in relation to total baseline and maximum cort levels. This model considers the effects of recent conditions (RC), ENSO, and energetic stores (ES) directly on CBG instead of inferring the relationship from the free cort model. From this model, we found several strong relationships between environmental variables and CBG levels for females and both environmental and energetic variables and CBG levels for males (Figure 4; Table A3). For females, we found negative relationships between RC and Total Baseline Cort (TBC) (*β* = −0.20; *υ* = 0.89), Total Maximum Cort (TMC) (*β* = −0.11; *υ* = 0.92), and CBG levels (*β* = −1.94; *υ* = 1), indicating lower levels across all components in response to improved recent conditions. Additionally, we found strong evidence for negative relationships between ENSO and TBC (*β* = −0.18; *υ* = 0.99) and CBG levels (*β* = −0.36; *υ* = 0.95), for females, indicating lower levels for both during unfavorable (wet) ENSO years. For males, we found strong evidence across all environmental and energetic variables on components of the CBG profile. We found strong evidence for the negative effects of ES on TBC (*β* = −0.24; *υ* = 1), TMC (*β* = −0.09; *υ* = 1), and CBG (*β* = −0.42; *υ* = 1), indicating lower levels across all components for individuals in better energetic condition. We also found positive effects of RC on TBC (*β* = 0.10; *υ* = 0.85) and CBG (*β* = 0.20; *υ* = 0.96), indicating higher baseline and CBG levels in response to improved recent conditions, as well as positive effects of ENSO on TMC (*β* = 0.03; *υ* = 0.90) and CBG (*β* = 0.33; *υ* = 1), indicating higher maximum and CBG levels in response to unfavorable ENSO years.

## 4 Discussion

Our study evaluated relationships between environmental conditions, energy stores, and corticosterone (cort) physiology in white‐crowned sparrows under three debated hypotheses. Comparisons of total and free hormone levels have been ongoing over the last 25 years, with varied support for total vs. free being the more biologically relevant measure (Mendel 1989; Breuner and Orchinik 2002; Malisch and Breuner 2010; Bonier et al. 2009). n this study we observed stronger and more numerous relationships when cort was characterized using the free hormone profile across environmental and energetic predictors. Some have argued that the relatively weak relationships observed in total hormone more closely reflect natural systems, implying that environmental and energetic conditions may not strongly predict circulating cort levels. Nevertheless, in our analysis relationships between cort physiology and both ENSO and energy stores were more consistently detected when examining free hormone dynamics. The CBG profile further helps explain some of the variation between the total and free models, especially in sex‐specific responses to ENSO (see below). Here, we first discuss differences between the three models and then explore the relationships discovered between cort physiology and both energy stores and ENSO.

**FIGURE 5:**
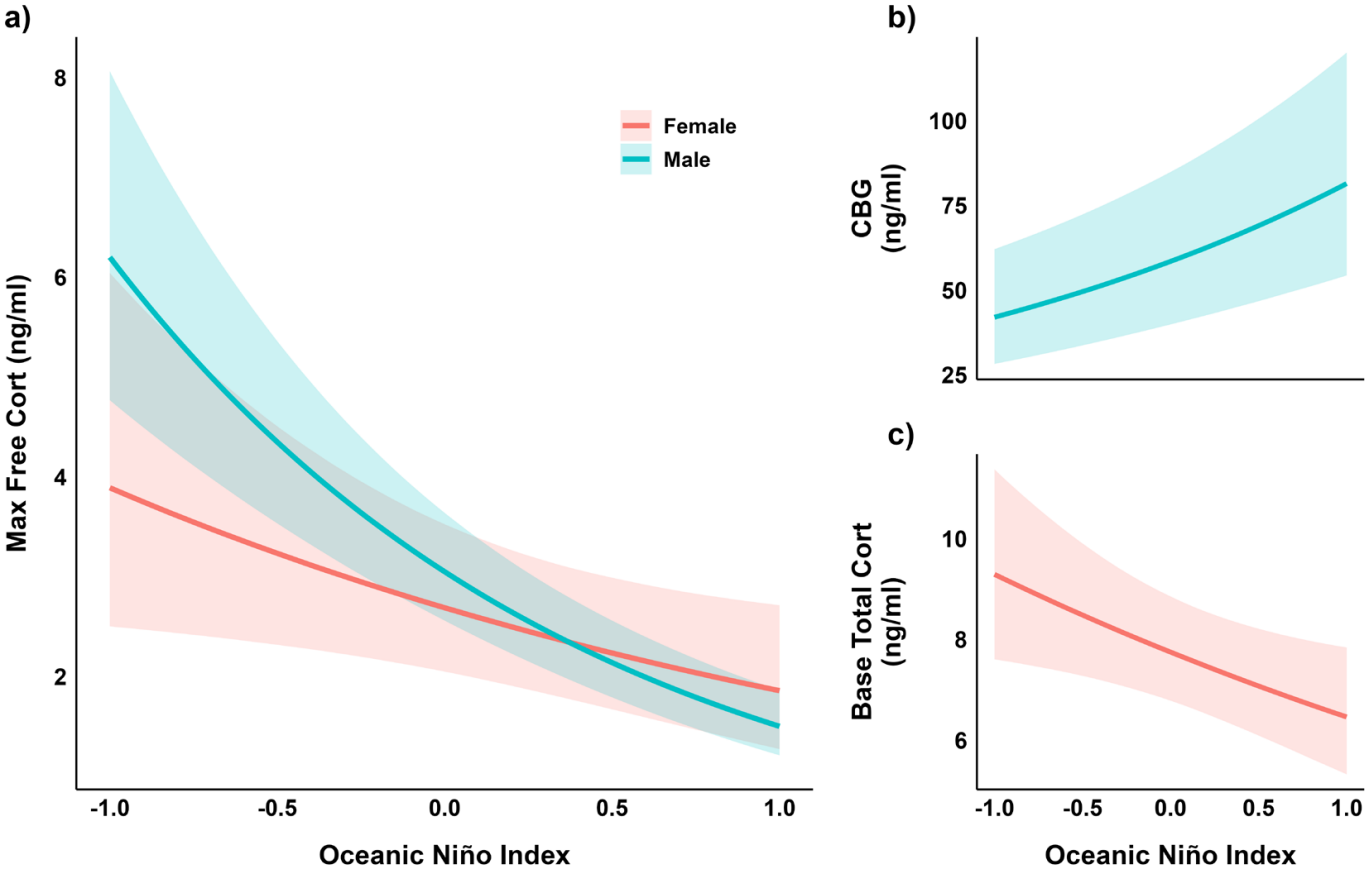
Declines in free cort with ENSO rely on different mechanisms in males and females. (a) Free cort levels decline with increasing Oceanic Nino Index for males (blue) and females (red). This results from (b) increasing CBG levels in males and (c) decreasing total cort levels in females. Data are taken from the CBG Profile Model.

**FIGURE 6:**
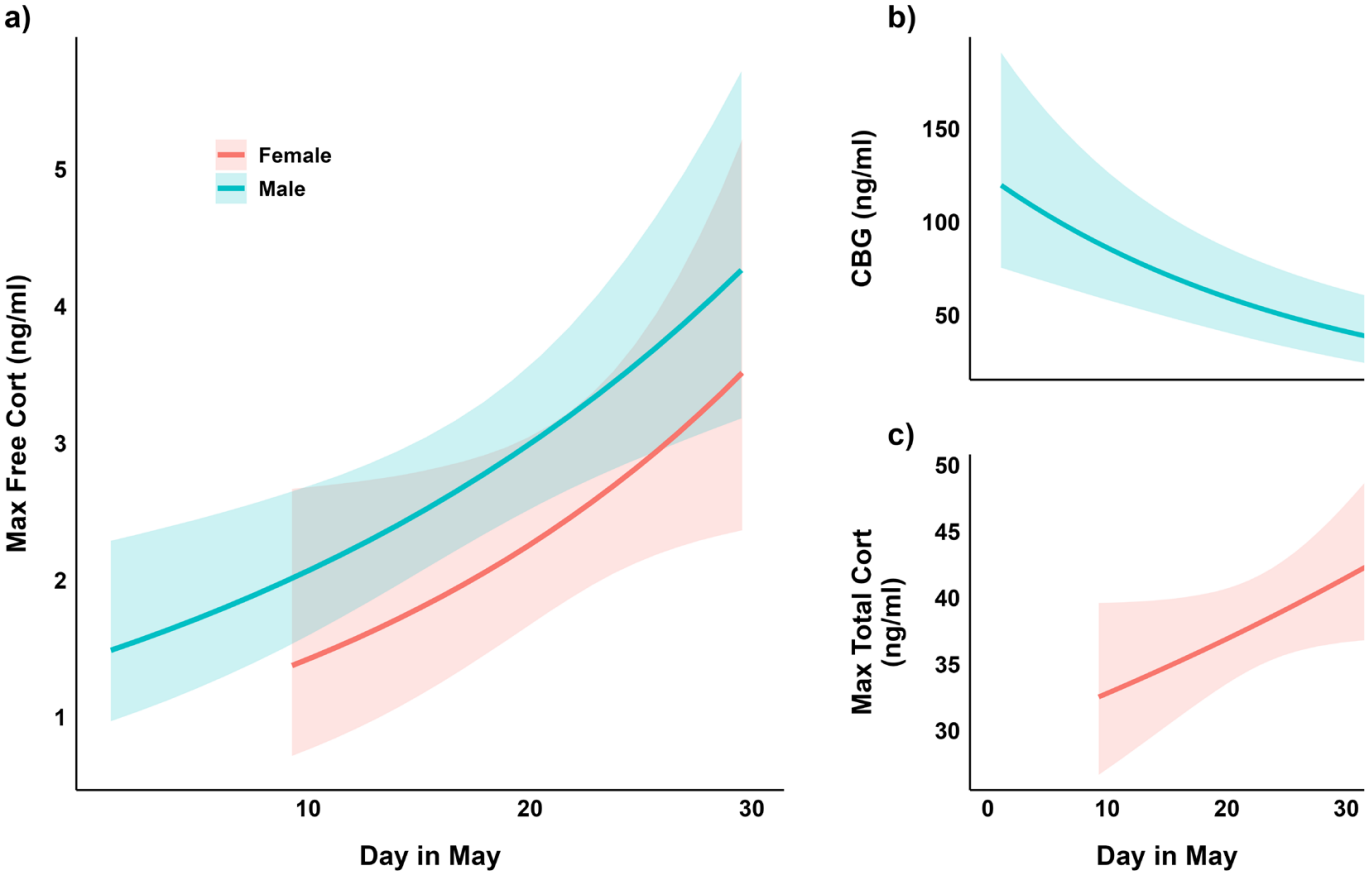
Increases in maximum free cort with date rely on different mechanisms in males and females. (a) Maximum free cort increases with date in males (blue) and females (red). This pattern emerges from a decline in CBG over that period in males (b), but an increase in total cort in females (c).

The “Total Hormone Profile” model exhibited the fewest relationships with our covariates, suggesting that total cort alone may have less explanatory power to capture the dynamic relationships between environment, energy, and cort (as suggested by Breuner, Delehanty, and Boonstra 2013). Total cort was significantly predicted by ENSO in females, and by energy stores in males. In contrast, the “Free Hormone Profile” revealed stronger and more numerous relationships. Notably, it indicated that free cort may better reflect hormonal regulation in response to environmental variability, independent of total hormone secretion (Breuner and Orchinik 2002). Specifically, free cort levels were strongly associated with ENSO in both sexes, providing compelling evidence of free cort's responsiveness to large‐scale climate patterns: Higher ENSO values (wetter years leading to later nest initiation) corresponded to decreased free baseline and free maximum cort levels (Figure 5a). Identification of these relationships supports free cort as a possible biomarker for response to climatic variability, at least for this population. The “Free Hormone Profile” also uncovered temporal differences in cort regulation. As the pre‐breeding period progresses through May, males and females increase both free baseline and maximum cort levels (Figure 6a). For research focusing on climate‐driven projections or temporal regulation of cort, our data suggest free cort is the more informative measure.

The “CBG Profile” model revealed the most detailed energy‐cort‐environment relationships, providing unique insights into sex‐specific cort regulation seen between total and free cort profiles. In particular, the model demonstrated that CBG may be a driving component for cort regulation, allowing males and females to regulate different components of their cort physiology to achieve similar outcomes. For instance, the shared negative relationship between wetter years and free cort levels was achieved via increased CBG levels in males (Figure 5b) but via reduced total levels in females (Figure 5c). Additionally, the pre‐breeding increases in free cort were attributed to decreased CBG levels in males (Figure 6b) but increased total levels in females (Figure 6c). Together these results demonstrate how reproductive timing and environmental conditions can uniquely shape hormonal regulation mechanisms in males and females. They also highlight the importance of downstream mediators as contributors to phenotypic variation in hormonal responses. For research focusing on mechanistic understandings of cort regulation or identifying potential targets of selection, the “CBG Profile” framework may offer particularly valuable insights.

### 4.1 Energy Stores and Cort Physiology

Glucocorticoid (GC) secretion is intricately connected to energy metabolism and behavioral regulation (Landys et al. 2006; Kirschman et al. 2017). Evidence suggests that GC physiology often reflects individual body condition and can indicate energy stores and metabolic demands (Krause et al. 2017; Jimeno et al. 2020). Baseline GCs regulate homeostatic energy balance by facilitating metabolism and stimulating foraging behavior with the energetic demands of predictable life events (Romero and Wingfield 2016). Consistent with this framework, individuals in poorer body condition typically exhibit elevated cort levels that promote resource acquisition and escape behavior (Angelier et al. 2009, Jaatinen et al. 2013; Breuner and Hahn 2003, Breuner et al. 1998; Wingfield and Kitaysky 2002). Our findings from the “Total Hormone Profile” and “CBG Profile” corroborate this expectation, revealing a negative relationship between energy stores and both max and baseline total cort levels in males.

Interestingly, the “Free Hormone Profile” did not align with these patterns. Free baseline cort did not reflect energy stores, and free maximum cort was positively associated with energy stores in males. These results suggest a more complex relationship between energetics and GC physiology, and the different patterns observed may reflect the need for flexibility in GC regulation in this high‐altitude population. The “Take it or Leave it” strategy (Wingfield et al. 1998) provides a useful framework for interpreting these results, especially considering the reservoir of cort bound to CBG in the plasma (Malisch and Breuner 2010). When food availability is sufficient, individuals may “take it” by maintaining most cort in the bound state while waiting out adverse conditions. However, food restriction is known to reduce CBG capacity in white‐crowned sparrows, thereby increasing free cort (Lynn et al. 2003). Under deteriorating environmental conditions, this shift could trigger a transition toward the “leave it” strategy, releasing much of the bound cort and eliciting a greater stress response to mobilize more extreme behavioral and physiological responses. Such flexibility may be especially important in unpredictable environments like the high Sierra Nevada, where sudden spring snowstorms can increase thermoregulatory costs and reduce foraging opportunities (Wingfield and Sapolsky 2003). The ability to modulate GC availability to tissues may therefore be critical for balancing immediate survival with long‐term fitness.

### 4.2 ENSO and Sex‐Specific Differences in Hormonal Regulation

Understanding how large‐scale climatic processes like the El Niño Southern Oscillation (ENSO) influence hormone patterns could become pivotal for predicting species' resilience to environmental stressors, particularly in the context of climate change (Wingfield et al. 2011; Cooke et al. 2013). There is relatively little known about the effects of large‐scale climatic events on GC physiology, likely due to the difficulty in collecting the breadth of data needed over an appropriate time frame (but see, Romero and Wikelski 2001; Wingfield et al. 2018). ENSO patterns cycle irregularly every two to seven years bringing changes in ocean temperature, air pressure, and rainfall trends (Trenberth 1997; NOAA). El Niño events are typically favorable for low‐elevation land birds, bringing increased precipitation and therefore plant productivity (Stenseth et al. 2002; Wingfield et al. 2018). In contrast, they are often unfavorable for sea birds as warm ocean waters reduce nutrient availability (Schreiber and Schreiber 1984; Ancona et al. 2011). The opposite occurs during La Niña events where increased ocean nutrients are favorable for seabirds and drought on land leads to unfavorable conditions for land birds (Stenseth et al. 2002). However, in high‐elevation systems across the Western US, El Niño's increased precipitation is associated with heavier snowfall (Cayan 1996), which delays the already short breeding seasons for ground‐ or shrub‐nesting birds (Martin and Wiebe 2004; Morton 1976). Thus, El Niño years are likely unfavorable for sparrow reproduction in the high Sierra, while La Niña years provide better environmental conditions and earlier breeding. Given our model specifications, we found that unfavorable, wetter ENSO years led to decrease baseline and maximum free cort levels in both male and female white‐crowned sparrows, while favorable drier years resulted in increased free cort levels. While the El Nino‐associated decrease in baseline free cort is consistent with delayed breeding under high snowpack—given that baseline cort typically increases with reproductive activity (Romero 2002; Bonier et al. 2009) ‐ the simultaneous reduction in stress‐induced cort contradicts current hypotheses (see below) and suggests sex‐specific regulation of free cort in response to ENSO, as discussed above (Breuner et al. Breuner, Delehanty, and Boonstra 2013; Love et al. 2004). Our results highlight the complex ways environmental variability affects GC physiology in sex‐specific ways.

High levels of cort may inhibit reproduction in favor of self‐preservation (Wingfield et al. 1998; Wingfield and Sapolsky 2003) and therefore modulate how organisms respond to “favorable” vs. “unfavorable” climatic events. We predicted reduced cort reactivity to challenge during favorable (dry) ENSO cycles to support reproduction under good environmental conditions and increased cort reactivity to challenge during unfavorable (wet) cycles to prioritize survival. For example, during an unfavorable El Niño period that caused famine, Galápagos marine iguanas exhibited increased baseline and maximum cort compared to a favorable La Niña period (Romero and Wikelski 2001). Similarly, high summer Pacific Decadal Oscillation values—an ENSO analog—were linked to increased cort levels, likely due to food scarcity in Rhinoceros auklets (Shimabukuro et al. 2023). However, the relationship between cort and ENSO does not always follow this pattern and can be mixed across species. In a comparative study of 12 Galápagos bird species, two land bird species exhibited suppressed cort levels during unfavorable ENSO years (Wingfield et al. 2018), while another study found this pattern in 7 out of 10 tropical birds (Messina et al. 2020). Sea lions also exhibited suppressed cort levels after a prolonged stretch of unfavorable El Niño conditions that reduced their nutritional state (DeRango et al. 2019). Our study falls in line with these latter examples, against our predictions, where cort physiology is suppressed during unfavorable years. Together, these findings across vertebrate taxa suggest that environmentally induced cort regulation is more nuanced than our current hypotheses support.

In our study system, we believe that higher free cort levels during favorable (dry) ENSO years may reflect an increase in flexibility for birds to abandon an unsuccessful breeding attempt in favor of a more promising one during lengthened (dry) breeding seasons. Conversely, lower levels during unfavorable (wet) years may act to buffer against poor environmental conditions by suppressing the full stress response during shortened reproductive cycles. Further, the “CBG Profile” model revealed sex‐specific mechanisms of free cort regulation in this response to ENSO. In males, total cort levels are high in unfavorable years, but CBG levels are also high, increasing the reservoir of cort in the blood. This strategy would allow individuals to maintain lower free cort levels until needed, providing flexibility in their stress response given the high likelihood of snow storms in wet El Niño years (Breuner et al. 2020). This pattern has also been seen in barn owl nestlings, where CBG capacity (total number present) was highest in a year of poor environmental conditions resulting in lower free cort levels than seen in a benign year (Almasi et al. 2009). Females keep total cort levels low, but arrive later in the season as the likelihood of storms is lower, and so the need for a reservoir is lower. Our findings may be the first to specify differences in sex‐specific mechanisms underlying hormonal responses to global climatic events and underscore the importance of assessing phenotypic plasticity in multiple components of the HPA‐axis when determining how environmental conditions impact endocrine responses.

## 5 Conclusions

Understanding how variation in cort profiles affects population dynamics could be a pivotal key for predicting population performance under future climate conditions. Equally important is determining how selection acts to shape this variation across individuals, populations, and species (Guindre‐Parker 2018). Hormonal regulation is complex, leading many to suggest that selection may be more likely to act on downstream effects or along multiple points of the HPA‐ axis than it is to act directly on total plasma concentrations (Hau et al. 2010). Different components of the endocrine system such as hormone receptors, enzymes, and binding proteins are all potential targets for selection and may contribute to phenotypic variations in hormonal responses between individuals, sexes, populations and species (Ketterson and Nolan, 1999). If variation in hormonal responses is a result of downstream mediators, using only total plasma concentrations will likely mask the effects (Breuner, Delehanty, and Boonstra 2013; Breuner and Orchinik 2002). In order to predict how cort levels within a population may be affected by future climatic, recent, and physiological conditions, we must first fully understand the mechanisms acting to moderate these levels, as selection may change these patterns over time.

Our findings highlight the complexity of environment‐cort‐energy dynamics in white‐crowned sparrows. While total cort provided some insights into these relationships, the free and CBG models revealed more nuanced and likely biologically relevant patterns, especially in the context of sex‐specific regulation and large‐scale climate drivers like ENSO. By illustrating how free cort and CBG levels mediate individual responses to environmental challenges, this study underscores the value of incorporating multiple components of endocrine regulation in future research. These insights have important implications not only for understanding the physiological strategies of wild populations but also for informing conservation efforts as climate change continues to impact species in dynamic and unpredictable ways. Future studies that integrate fitness outcomes with hormone dynamics will be crucial in refining our understanding of how these physiological mechanisms shape survival and reproduction in changing environments.

## Author Contributions

**B. Sunny Domschot:** conceptualization (equal), formal analysis (equal), investigation (equal), methodology (equal), visualization (equal), writing – original draft (equal), writing – review and editing (equal). **Thomas V. Riecke:** formal analysis (equal), methodology (equal). **Jessica L. Malisch:** data curation (equal), writing – review and editing (equal). **Thomas P. Hahn:** data curation (equal), writing – review and editing (equal). **Creagh W. Breuner:** data curation (equal), resources (equal), supervision (equal), writing – original draft (equal), writing – review and editing (equal).

## Funding

This work was supported by the NSF grants PSI‐0747361 to C.W.B. and IBN‐0236536 to C.W.B. and T.P.H. Addition support was provided by a fellowship from the American Association of University Women.

## Conflicts of Interest

The authors declare no conflicts of interest.

## Supporting information


**Table A1.** The total hormone profile hypothesis model results.
**Table A2**. The free hormone profile hypothesis model results.
**Table A3**. The CORT profile hypothesis model results.

## Data Availability

All the required data are uploaded as Supporting Information S1.
