# Supplementary material for: Corticosterone as a Physiological Biomarker: Decoding the Environment‐Cort‐Energy Paradigm
Source: Ecol Evol. 2026 Apr 29;16(5):e73572. doi: 10.1002/ece3.73572 (PMC13126097; doi:10.1002/ece3.73572)
Supplement: Supplementary file 1 — Table A1. The total hormone profile hypothesis model results. Table A2. The free hormone profile hypothesis model results. Table A3. The CORT profile hypothesis model results. [file ECE3-16-e73572-s001.docx]

**APPENDIX**

**Tables A1-A3.** Beta and F-Values for covariate effects on CORT levels under each hypothesis. Colored boxes indicate a positive (blue) or negative (red) covariate effect known with either 95% (dark fill & bold font) or 85% (light fill & non-bold font) confidence.

**Table A1.** The Total Hormone Profile Hypothesis Model Results

|  | Females | | | | Males | | | |
| --- | --- | --- | --- | --- | --- | --- | --- | --- |
|  | Total Base | | Total Max | | Total Base | | Total Max | |
|  | β | F | β | F | β | F | β | F |
| Energy Stores | -0.13 | 0.92 | 0.00 | 0.53 | **-0.25** | 1.00 | **-0.11** | 1.00 |
| Recent Cond. | 0.12 | 0.79 | 0.06 | 0.74 | 0.07 | 0.83 | 0.04 | 0.81 |
| ENSO | **-0.16** | 0.99 | -0.05 | 0.90 | -0.02 | 0.73 | 0.02 | 0.82 |
| Time | **0.15** | 0.97 | 0.04 | 0.78 | 0.06 | 0.93 | -0.01 | 0.74 |
| Day | 0.02 | 0.61 | 0.05 | 0.89 | 0.03 | 0.75 | 0.02 | 0.78 |
| Base |  |  | **0.01** | 1.00 |  |  | **0.01** | 1.00 |

**Table A2.** The Free Hormone Profile Hypothesis Model Results

|  | Females | | | | Males | | | |
| --- | --- | --- | --- | --- | --- | --- | --- | --- |
|  | Free Base | | Free Max | | Free Base | | Free Max | |
|  | β | F | β | F | β | F | β | F |
| Energy Stores | 0.61 | 0.85 | 0.18 | 0.83 | 0.12 | 0.64 | **0.18** | 0.97 |
| Recent Cond. | -0.76 | 0.85 | 0.26 | 0.85 | 0.26 | 0.72 | 0.02 | 0.54 |
| ENSO | **-1.38** | 1.00 | **-0.37** | 0.99 | **-1.93** | 1.00 | **-0.71** | 1.00 |
| Time | 0.13 | 0.60 | 0.11 | 0.75 | -0.02 | 0.53 | 0.01 | 0.55 |
| Day | **1.25** | 1.00 | **0.27** | 0.98 | **0.86** | 1.00 | **0.29** | 1.00 |
| Base |  |  | **0.48** | 1.00 |  |  | **0.18** | 1.00 |

**Table A3.** The CORT Profile Hypothesis Model Results

|  | Females | | | | | | Males | | | | | |
| --- | --- | --- | --- | --- | --- | --- | --- | --- | --- | --- | --- | --- |
|  | Total Base | | Total Max | | CBG | | Total Base | | Total Max | | CBG | |
|  | β | F | β | F | β | F | β | F | β | F | β | F |
| Energy Stores | -0.01 | 0.54 | 0.04 | 0.78 | 0.04 | 0.54 | **-0.24** | 1.00 | **-0.09** | 0.99 | **-0.42** | 1.00 |
| Recent Cond. | -0.20 | 0.89 | **-0.11** | 0.92 | **-1.94** | 1.00 | 0.10 | 0.85 | 0.02 | 0.65 | **0.20** | 0.96 |
| ENSO | **-0.18** | 0.99 | -0.03 | 0.75 | **-0.36** | 0.95 | -0.02 | 0.69 | 0.03 | 0.90 | **0.33** | 1.00 |
| Time | 0.11 | 0.92 | 0.01 | 0.61 | -0.01 | 0.52 | 0.02 | 0.68 | -0.02 | 0.77 | **0.08** | 0.96 |
| Day | 0.07 | 0.85 | **0.07** | 0.97 | **-0.59** | 1.00 | **0.09** | 0.99 | 0.03 | 0.86 | **-0.29** | 1.00 |
| Base |  |  | **0.01** | 1.00 | -0.04 | 0.82 |  |  | **0.02** | 1.00 | 0.00 | 0.57 |
| Max |  |  |  |  | 0.01 | 0.78 |  |  |  |  | **0.01** | 1.00 |
